# THE MAIN CYTOTOXIC EFFECTS OF METHYLSELENINIC ACID ON VARIOUS CANCER CELLS

**DOI:** 10.3390/ijms22126614

**Published:** 2021-06-21

**Authors:** Elena G. Varlamova, Egor A. Turovsky

**Affiliations:** Institute of Cell Biophysics of the Russian Academy of Sciences, Federal Research Center “Pushchino Scientific Center for Biological Research of the Russian Academy of Sciences”, Institutskaya St. 3, Pushchino 142290, Moscow Region, Russia; turovsky.84@mail.ru

**Keywords:** methylseleninic acid, cytotoxicity, cancer, selenoproteins

## Abstract

Studies of recent decades have repeatedly demonstrated the cytotoxic effect of selenium-containing compounds on cancer cells of various origins. Particular attention in these studies is paid to methylseleninic acid, a widespread selenium-containing compound of organic nature, for several reasons: it has a selective cytotoxic effect on cancer cells, it is cytotoxic in small doses, it is able to generate methylselenol, excluding the action of the enzyme β-lyase. All these qualities make methylseleninic acid an attractive substrate for the production of anticancer drugs on its basis with a well-pronounced selective effect. However, the studies available to date indicate that there is no strictly specific molecular mechanism of its cytotoxic effect in relation to different cancer cell lines and cancer models. This review contains generalized information on the dose- and time-dependent regulation of the toxic effect of methylseleninic acid on the proliferative properties of a number of cancer cell lines. In addition, special attention in this review is paid to the influence of this selenium-containing compound on the regulation of endoplasmic reticulum stress and on the expression of seven selenoproteins, which are localized in the endoplasmic reticulum.

## 1. Introduction

It is well known that the toxicity of selenium and its compounds largely depends on their chemical form and dose. It is believed that inorganic selenium species are more toxic, and the concentration range between insufficient and excess intake is quite narrow [1]. Studies of recent decades have repeatedly demonstrated the antitumor activity of selenium-containing compounds of various natures [2,3,4,5,6,7,8,9,10]. All selenium drugs exhibiting antitumor activity can be divided into two groups: generators of hydrogen selenide and methylselenol, which have pronounced toxic properties. For this reason, the study of existing and new selenium-containing drugs with antitumor activity should be directed to those molecules that can generate hydrogen selenide or methylselenol. Methylselenol (CH_3_SeH) is a key metabolite in the anticancer activity of selenium compounds; however, the in situ production or, alternatively, the use of precursors is required due to the high reactivity and volatility of this molecule. Methylselenol is more biologically active than its analogs: selenol, etanselenol, 2-propanselenol [11,12]. Selenols are organic compounds that contain a functional group (CSeH) and are often called selenothiols. They play an important role in biological processes, being part of the active centers of a number of enzymes: glutathione peroxidases, thioredoxin reductases, iodothyronine deiodinases. Selenols are obtained by the reaction of organic lithium reagents and Grignard reagents with elemental selenium [13]. Because selenols are readily oxidized to diselenides, they are rarely used as metabolites in cancer prevention studies.

The most studied precursors of methylselenol are methylselenocysteine (MSC), selenomethionine (SM), and methylselenic acid (MSA). So SM is metabolized through a multistage transsulfurization pathway to selenocysteine (Sec), which is first degraded to hydrogen selenide. Further, the reaction of methylation of hydrogen selenide by the enzyme methyltransferase to methylselenol occurs [14].

MSC, as a methylselenol generator, has several advantages over SM. First, MSC is cleaved in one step to methylselenol by the enzyme β-lyase. Second, MSC accumulates in tissues to a lesser extent and is eliminated from the body faster than SM [15,16], which can be nonspecifically incorporated into selenoproteins instead of selenomethionine, which makes it less available for further metabolism [17]. A significant disadvantage of using MSC as a precursor of methylselenol is that in some cancer cells, the activity of the β-lyase is quite low, which leads to an increase in the amount of MSC in the medium, the concentration of which significantly exceeds its physiological concentration in plasma (3–5 μM). This, in turn, leads to the nonspecific effects of MSC, which are by no means always antitumor [14].

**Figure 1:**
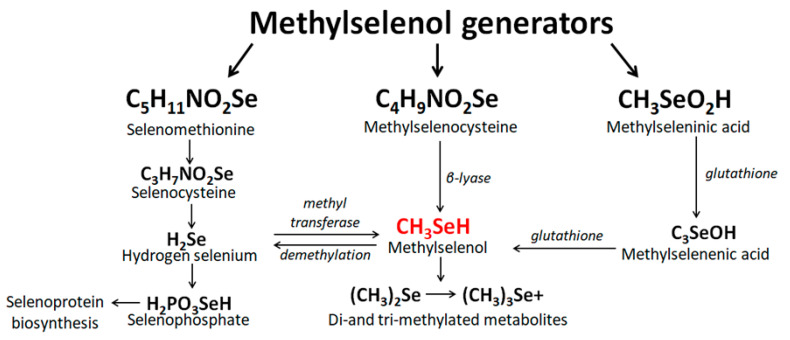
The conversion reactions of the most common methylselenol generators.

It is believed that MSA is a reagent for endogenous formation of methylselenol in a simple stoichiometric manner immediately after entering cells, where it is easily reduced to methylselenol through non-enzymatic and enzymatic reactions involving glutathione (GSH) and NADPH [18]. In addition, using yeast as an example, it was shown that methylselenol, in addition to MSA, can be efficiently produced from methylselenoglutathione (MSSG) and dimethyldiselenide (DMDS), provided that the ratio GSH/GSSG in cells is sufficiently high [19]. Interestingly, the inhibitory concentration of MSA with respect to the BY4742 strain of *Saccharomyces cerevisiae* decreased by an order of magnitude when the cells were exposed to complex MSA/GSH or MSA/DMDS, compared to the treatment of cells with MSA alone. The authors suggest that this effect may be associated with an increase in total selenium in cells (approximately 15 times) compared to the effect on cells only of MSA. According to the authors, methylselenol is toxic to yeast since it is metabolized to SM, which causes the aggregation of toxic proteins [19]. They showed this in a mutant form of *Saccharomyces cerevisiae* (met17) that lacked the enzyme O-acetylhomoserine, which catalyzes the conversion of methylselenol to selenomethionine. However, this enzyme is absent in higher eukaryotes; therefore, the authors used this mutant strain to search for alternative targets for methylselenol [20]. It has been shown that DMDS prevents the formation of a disulfide bond in carboxypeptidase Y, contributing to the retention of the proenzyme in the ER. Thus, the authors hypothesized that DMDS, which is an oxidized compound, contributes to the reductive stress in the ER. Most likely, most of DMDS is converted to methylselenol in the cytosol, and the subsequent diffusion of methylselenol into the ER can increase the ratio GSH/GSSG in the ER and lead to reductive stress in this compartment. Figure 1 schematically shows the main mechanisms for obtaining methylselenol from its precursors.

This review focuses on one of the important recursors of methylselenol-MSA. Here we describe the experimental data of recent years devoted to the study of the cytotoxic mechanisms of MSA in various mammalian cancer cells, its role in the regulation of carcinogenesis and ER stress. Among all known organic selenium-containing compounds, MSA occupies a special place. Firstly, it is an organic and low-toxic compound, which is a generator of methylselenol. Even small concentrations of MSA in the nutrient medium are capable of causing adaptive ER stress, which does not lead to cell death. Secondly, due to the selective cytotoxicity of its action, it is possible to select such concentrations of MSA that lead to prolonged ER stress and death of cancer cells but do not affect the viability of normal cells, which is very important in cancer therapy. However, until now, the growth, modulating, and cytotoxic mechanisms of MSA influence are poorly understood. In order to get closer to understanding the causes of the cytotoxic effect of MSA on cancer cells, it is necessary to consider this problem in a multifaceted manner. A special role should be given to the mechanisms of regulation of endoplasmic reticulum (ER) stress (ER stress), activation of signaling pathways of adaptive and pro-apoptotic stress, as well as the effect on the expression and activity of seven selenoproteins localized in the ER and taking an active part in the processes of carcinogenesis and ER stress [21,22,23,24,25].

## 2. Reasons and Molecular Mechanisms of the Cytotoxic Effect of MSA on Cancer Cells

The mechanism of action of selenium-containing compounds, including MSA, on the proliferative properties of cancer cells, is explained by a number of factors. First, it is known that the cell membranes of cancer cells contain a large number of sulfhydryl groups that form disulfide bonds with fibrinogen polypeptide chains, which leads to the formation of a high molecular weight polymer similar to fibrin-parafibrin [26,27,28]. Parafibrin is resistant to proteolytic degradation, forms a “shell” on the surface of cancer cells, protecting them from destruction by phagocytic cells. The decrease in the proliferative properties of cancer cells can be explained by the action of selenium-containing compounds as inhibitors of sulfhydryl groups.

On the other hand, MSA enhances the generation of reactive oxygen species (ROS) by cancer cells [29], also leads to depletion of intracellular glutathione and, thus, the cellular environment becomes more oxidized, which can cause cell death. It has been repeatedly shown that glutathione promotes the resistance of cancer cells to various anticancer drugs by covalent binding and subsequent inactivation of these drugs [18]. On the example of human lung cancer cells (line A549), it was shown that glutathione was crucial for the metabolism of MSA [30]. MSA nonenzymatically metabolized glutathione, which was ubiquitous in cancer cells. Lui et al. suggested that MSA is converted by glutathione into methylselenol, which is then demethylated to hydrogen selenide, which is an important ROS generator [31].

**Figure 2:**
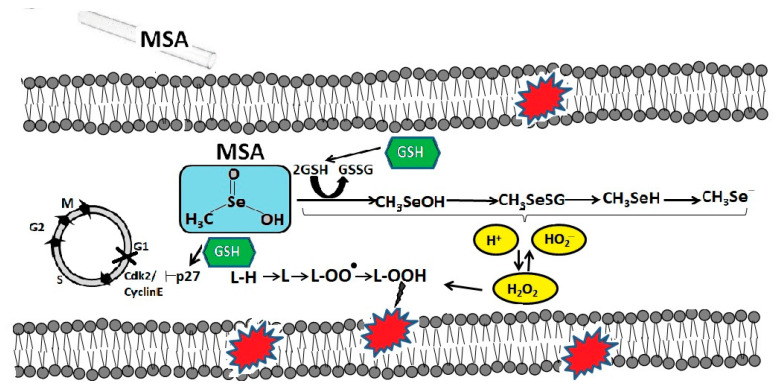
The molecular mechanisms of MSA cytotoxicity mediated by intracellular glutathione in cancer cells. Reduced glutathione (GSH), present in cancer cells in large amounts, is extremely important for the metabolism of MSA. In the process of converting MSA into methylselenol, glutathione is oxidized (GSSG) and free radicals, in particular, hydrogen peroxide, increase, which are the cause of the oxidative degradation of membrane lipids. In addition, MSA, through the oxidation of GSH, contributes to the G1 arrest of the cell cycle by suppressing the activity of cyclin E1 and activating the cyclin-dependent kinase 2 inhibitor p27Kip1.

It has been suggested that intracellular glutathione may play a key role in cell cycle arrest and MSA-induced apoptosis [32]. It is assumed that MSA induced G1 arrest by down-regulating cyclin E1 and up-regulating p27Kip1 (cyclin-dependent kinase 1B inhibitor). It has also been shown that human hepatoma HepG2 cells with a high concentration of glutathione are more sensitive to MSA [33]. In addition, it was found that MSA makes cancer cells more sensitive to radiation and causes their toxicity through glutathione-dependent induction of lipid peroxidation, which was demonstrated by the example of head and neck squamous carcinoma cell lines [34]. Possibly, the selective cytotoxicity of MSA against cancer cells can be explained by the higher lipid content in cancer cells compared to normal cells [35,36,37,38,39]. On the example of a line of human monocytic cells obtained in acute monocytic leukemia (THP-1 line), it was shown that the concentration of glutathione in them is 40 times higher than that in mononuclear cells of peripheral blood. When these cells were treated with MSA at concentrations from 2.5 to 15 μM for 6 h, a significant decrease in glutathione levels in THP-1 cancer cells and a dose-dependent increase in its concentration in normal PBMS cells were observed [40]. Thus, the simultaneous increase in glutathione in normal cells and its depletion in cancer cells can contribute to the improvement of cancer therapy by reducing the toxicity of normal tissues and enhancing the antitumor effect of the drug on a malignant tumor. The molecular mechanisms of MSA cytotoxicity mediated by intracellular glutathione in cancer cells are schematically shown in Figure 2.

It has been shown that MSA effectively inhibited angiogenesis. Thus, it was shown on the model of human umbilical vein endothelial cells (HUVEC) that exogenous MSA was not only able to maintain the level of nutrient selenium but also effectively inhibited cell migration and neoangiogenesis by suppressing β3-integrin and interrupting its clustering, as well as by inhibiting phosphorylation of AKT, IkBα and NF-kB [41]. Integrins are known to play an important role in cell adhesion, migration, and signal transduction; however, MSA suppressed the expression of only β3-subunit of integrin and did not affect the expression of αv, α1, α5, β1, and β5. It is known that β3-subunits of integrin are largely expressed in endothelial cells, during angiogenesis conformational changes and clustering of β3-integrin is observed, which initiates intracellular signal transduction through the phosphorylation cascade of FAK, AKT, IKKβ, IκBα, and NF-κB [42,43]. In addition, β3-integrin during angiogenesis regulates the expression of cytokines (TNFα, IL-1β, IL-6), adhesion molecules (VCAM-1, ICAM-1, E-selectin), enzymes (iNOS, COX-2) [44,45,46,47], and also regulates cell polarity and their directional migration [48,49]. 

One of the important effects of MSA on cancer cells is the activation of FOXO proteins by phosphorylation, which ultimately triggers the signaling PI3K/AKT/mTOR (phosphoinositide 3-kinase/protein kinase B/mechanistic target of rapamycin) pathway-is an intracellular signaling pathway important in regulating the cell cycle [50,51,52,53,54]. For example, using A549 cancer cell line as an example, it was shown that 5 μM MSA already 1.5 h after the start of cell treatment promoted dephosphorylation of FOXO 3a and its translocation into the nucleus, and, consequently, induction of apoptosis, suppression of the viability of cancer cells and G-1 arrest of the cell cycle. Thus, it was shown that FOXO 3a is an important mediator of the antitumor action of MSA [54]. In addition, 5 μM MSA increased the expression of the pro-apoptotic Bax gene and cytosolic cytochrome C, decreased the level of procaspase-3, and promoted the PARP release. It was also shown that MSA in A549 cells blocked glycolysis, the tricarboxylic acid cycle, and nucleotide biosynthesis since the levels of lactate, malate, aspartate, glutamate, citrate, as well as adenine and uracil, decreased in the treated cells [54].

In the example of MC38 murine intestinal cancer cells and colorectal cancer xenograft models, it was shown that MSA suppressed the growth of tumor cells by activating caspase-3 but did not affect the level of active p53 protein. In addition, MSA increased the selenium content in the liver, kidneys, muscles, stomach, and plasma, and also increased the activity of glutathione peroxidases in the blood of model animals, without affecting the muscle and fat composition of the body, the level of leptin and adinopectin in plasma. MSA (3 mg/kg body weight) inhibited tumor growth by up to 61% compared with the control group, which was associated with a decrease in the levels of tumor necrosis factor TNFα and interleukin 6 (IL6) [55]. In addition, MSA, especially at concentrations of 5 and 15 μM, was capable of activating caspase-8 in THP-1 cancer cells and, conversely, inhibiting them in healthy PBMS cells, which is consistent with previously obtained data that selenium compounds in a dose-dependent manner were able to induce apoptosis in cancer cells while protecting normal tissues [56,57,58].

Using 4T1 mouse malignant breast cancer cells as an example, it was shown that MSA significantly induced apoptosis of these cancer cells by activating Bax, caspase-3, PARP [59]. In addition, MSA was able to inhibit tumor angiogenesis by reducing the expression of vascular endothelial growth factors VEGF and Ang-2 in mammary cells of dogs and mouse models. This series of experiments showed that MSA inhibited the JAK2/STAT3 signaling pathway [60].

It has been shown that MSA also activated the Keap1/Nrf2 pathway in ESCC cell lines: KYSE 150, KYSE 410, KYSE 180, and KYSE 510 [32]. The Keap1-Nrf2 (kelch-like ECH-associated protein 1/nuclear factor E2-related factor) pathway is known to be one of the main protective responses of the cell to oxidative and electrophilic voltages. Under homeostatic conditions, Keap1 is part of E3-ubiquitin ligase, which regulates the activity of the transcription factor Nrf2, directing it to ubiquitination and proteasome-dependent degradation. A complex molecular mechanism is triggered under stress conditions, in which sensory cysteines inside Keap1 allow to avoid ubiquitination of Nrf2, and promote its accumulation in the cell and transfer to the nucleus, where it can start its antioxidant transcription program [61]. The treatment of ESCC cell lines with MSA has been shown to significantly suppress Keap1 both in the nucleus and in the cytoplasm and enhance the expression of Nrf2 and its concentration in the cell nucleus. In addition, MSA activated the Keap1/Nrf2 pathway via upregulation of miR-200a. Thus, when ESCC cells were treated with 5 μM MYF for 24 h, a significant induction of miR-200a expression was observed. Thus, Keap1 is a direct target of miR-200a in ESCC cells [32].

WM1552c, UKRV, Colo875 (human melanoma cell lines), SK-BR-3, BT-474 (human mammary carcinoma cell lines), B16F10 (mouse skin melanoma cells) treatment with MSA increased the MHC class I (major histocompatibility complex surface) expression levels in all the tested tumor cell lines [62]. The human MHC class I molecules present antigenic peptides on the surface of cells, which are predominantly generated by proteasomal degradation of intracellular proteins. MSA partially modulates IFNγ (interferon gamma) signaling, such as the upregulation of STAT1 (Stat1 signal transducer and activator of transcription 1), JAK1 (Janus kinase 1), IRF1, IRF5, IRF7, and IRF9 (Interferon-regulated factor1, 5, 7 and 9) on the mRNA and/or protein expression levels. In addition, MSA treatment resulted in activation of the transcription factor Nrf2 (nuclear factor erythroid 2-related factor 2).

MSA can be the cause of another variant of cell death, entosis. Entosis is a type of programmed cell death in which one epithelial cell is absorbed by another epithelial cell and subsequently dies in the vacuole or lysosome of the absorbed cell. Entosis was first described under anchorage-independent conditions and the loss of β1-integrin (CD29) signaling [63]. For example, on Panc-1 (human pancreatic ductal adenocarcinoma) cells, it was shown that MSA induced entosis by cell detachment through downregulation of cell division control protein 42 homologs (CDC42) and its downstream effector β1-integrin (CD29) [64]. Treatment with MSA led to a unique phenotype, characterized by changes in morphology and cell detachment from the culture plate prior to cell death. Detached cells were alive at 24 h, but their reattachment capability and colony-forming ability had been dramatically compromised.

Treatment of Eca109 cells (human esophageal carcinoma cell line) with 20 and 40 μM MSA for 72 h reduced cell viability by only approximately 50% and with 20 μM MSA for 48 h cell cycle was arrested in G0/G1 phase, and cell population was increased in S phase. It was found that MSA significantly reduced the expression of FAL1 (focally amplified lncRNA on chromosome 1), and thus, the level of PTEN (phosphatase and tensin homolog deleted on chromosome 10) was increased; therefore, PTEN is a target for FAL1 [65]. It is known that PTEN-phosphatase has dual substrate specificity, which catalyzes the cleavage of the phosphate group at the position of the 3D inositol ring of phosphatidylinositol-3-phosphates, thus depriving them of the functions of secondary messengers in signal transduction in the cell. This phosphatase is one of the few negative regulators of the PI3K/AKT/mTOR signaling pathway.

Special attention should be paid to another important effect of selenium on the prevention of cancer development-epigenetic modification of the genome, in particular, deacetylation of histones, as was shown in the example of human breast adenocarcinoma (MCF7 cell line) [66], and also contributes to DNA methylation [67]. It was shown that MSA was able to inhibit the activity of histone deacetylase, which plays an important role in the regulation of the expression of important genes, promoting the modification of histones and changing the chromatin conformation [68]. In MCF7 cells, MSA inhibited the expression of DNA methyltransferase 1 (DNMT1) and also influenced certain histone labels, increasing H4K16ac and decreasing the level of H3K9me3 [66].

In addition, it has been shown that the target for MSA is the hypoxia-inducible transcription factor 1α (HIF-1α) in cancer cells under hypoxic conditions [69]. It is known that hypoxic tumor cells, such as human head and neck squamous cell carcinoma (HNSCC), overexpress the HIF-1α. This makes cancer cells resistant to chemotherapy and radiation therapy [70,71]. The factor is stabilized under hypoxia, which occurs due to the inhibition of prolylhydroxylases (PHDs) that hydroxylate proline molecules of HIF-1α, leading to ubiquitylation by von Hippel-Lindau protein (VHL) and degradation by proteosomes [72]. Under normoxia, the ubiquitin protease-mediated pathway rapidly destroys factor molecules. It was shown that MSA effectively inhibits the HIF-1α in hypoxic cells, while PHD 2 and PHD 3, on the contrary, were activated, which was demonstrated in HNSCC cells [73]. In addition, a decrease in the ROS level was observed, comparable to the nomoxic controls, which was also accompanied by the stabilization of prolylhydroxylases. The resistance of these cancer cells, overexpressing the HIF-1α under hypoxia, to SN38-the active metabolite of irinotecan, was reduced after MSA treatment. Similar synergistic activity of MSA in combination with docetaxel was shown in a model of prostate cancer cells [74], which can also be explained by the inhibition of HIF-1α by the activation of PHDs [75,76,77].

Treatment of clear cell renal cell carcinoma (RC2 and 786-0) with a pharmacological dose of MSA (10 μM) promoted inhibition of constitutively expressed transcription factors HIF-1α and HIF-2α in RC2 and 786-0 cells, respectively [78]. In RC2 cells, this was caused by suppression of activity VHL, but not in 786-0 cells. However, under hypoxia, when the secretion of VHL is increased in comparison with normoxia, MSA is able to inhibit this secretion. It was also shown that the degradation of HIF-1α after MSA treatment does not depend on VHL but depends on PHD2. Thus, MSA cannot degrade HIF-1α stabilized by a DMOG (dimethyloxallyl glycine)-inhibitor of PHD activity.

It has been shown that MSA inhibits the expression and activity of HIF-1α in invasive rat and human prostate cancer cells [79]. Thus, the treatment of highly aggressive human prostate cancer cell lines (PC-3 and PC-3M) with MSA led to significant inhibition of growth and induction of apoptosis, and MSA has a stronger effect on cells under hypoxia than under normoxia, especially at a physiological dose of MSA (5 μM). These data suggest that MSA-induced apoptosis under hypoxia is not unique to PTEN-mutant (PC-3 and PC-3M) or PTEN-positive cells (PAIII and DU145). At the same time, a decrease in the expression of the HIF-1α protein in PAIII and PC-3M cells was also observed in a dose-dependent manner. However, PC-3M cells in the presence of serum appeared to be more resistant to MSA treatment under hypoxia, suggesting that growth factor (serum)-induced signals such as PI3K (phosphoinositide 3-kinase), IGF-1 (insulin-like growth factor-1), or EGFR (epidermal growth factor receptor) may impart partial resistance. DNA binding of HIF-1α was significantly reduced by MSA in PAIII and PC-3 cells in both normoxia and hypoxia. In addition, HRE (hypoxia-response element) activity during hypoxia in PC-3M cells was reduced after MSA treatment. Other selenium compounds, including SM, MSC, and selenite, showed no significant changes in HRE activity, suggesting that MSA, possibly acting as a precursor of methylselenol, exhibits specificity for redox proteins, especially under conditions of hypoxia. Treatment of prostate cancer cells with MSA also reduced the expression of the VEGF and GLUT 1 genes. Both VEGF (vascular endothelial growth factor) and GLUT 1 (glucose transporter 1) are downstream targets of HIF-1α and play an important role in HIF-1α-induced cancer invasion [80]. It is known that CoCl 2 (cobalt chloride) mimics hypoxia under normoxia and thus induces HIF-1α [81]. When prostate cancer cells were either pretreated with MSA followed by CoCl 2 or co-treated with MSA and CoCl 2, there was a marked decrease in HIF-1α binding or protein expression, respectively. These observations collectively indicate the efficacy of MSA against invasive prostate cancer growth that occurs under hypoxia. These results can be applied to clinical therapies since the hypoxic microenvironment in solid tumors correlates with tumor invasiveness, metastasis, and drug and radiation resistance.

## 3. The Role of MSA in the Regulation of ER-Stress

ER is an extensive membrane organelle that plays an essential role in the viability of a eukaryotic cell. The main function of granular ER is participation in the synthesis and folding of proteins intended for secretion or exposure on the surface of the cell membrane. For proper folding of a protein molecule, including glycosylation, phosphorylation, hydroxylation, and other modifications, conditions close to the characteristics of the extracellular environment are necessary. A situation in which the intermediate form turns out to be so unfortunate that it leads to unintended interactions with cellular components is called misfolding or folding error, which poses a significant threat to the cell and the body as a whole and is the cause of ER stress. ER stress is a molecular pathophysiological process underlying many human diseases, and impaired protein folding is important for its development. To prevent this situation, eukaryotes have developed a complex homeostatic mechanism known as the unfolded protein response (UPR). In the event that the UPR efforts are unsuccessful and ER-stress deepens, the main UPR regulators are incorporated into the apoptosis signaling cascade.

Many studies have repeatedly demonstrated that MSA is one of the main selenium nature sources of ER stress, and the mechanisms of ER stress regulation are rather ambiguous for different cell lines and tumor models [3,4,8,10,82]. When analyzing a large number of works devoted to this topic, it becomes clear that MSA in low concentrations can only lead to an adaptive response of cells to ER-stress, without causing apoptosis, while higher concentrations of MSA (0.1–1 μM and higher) becomes destructive for a number of cancer cell lines, causing acute ER-stress and apoptosis [3,4,8]. In response to the treatment of cells with MSA, various UPR signaling pathways are triggered, which indicates the absence of a specific mechanism for the regulation of ER stress. Most likely, MSA disrupts redox homeostasis in cells, but depending on the sensitivity of a particular cell line to this inducer and the general toxic effect caused by it, one or another signaling cascade is triggered, and sometimes several at once.

We have previously shown [3] that in human prostate adenocarcinoma cells (DU 145), MSA promoted the activation of the PERK signaling pathway since an increase in ATF-4 expression was found. In addition, an increased expression of apoptosis genes, as well as effector caspase-3 and inflammatory caspase-4, was observed in these cancer cells, which may indicate activation of the pro-apoptotic caspase pathway. According to the results of real-time PCR and quantitative assessment of proteins in the studied samples, in human fibrosarcoma cells (line HT-1080), MSA, most likely, promoted the activation of the ATF-6 signaling pathway.

In MCF 7 cells, MSA simultaneously activated two pro-apoptotic signaling pathways UPR: IRE1 and ATF-6. Such an interconnection of signaling pathways is well known: post-translational activation of ATF-6 promotes the activation of post-transcriptional modification (excision of the 26-nucleotide intron) from the unspliced XBP1u, which may serve as an additional explanation for the activation of both signaling pathways.

In addition, we previously established that when mouse testicle teratocarcinoma cells (line F-9) were treated with 1 μM MSA for 24 h, the expression of the spliced XBP1s form increased significantly, which may indicate activation of the IRE1-signaling pathway [4].

On cells of primary effusion lymphoma (PEL line), it was shown that MSA activated caspase-4 and genes of pro-apoptotic and apoptotic UPR: GRP78, XBP1s, CHOP, GADD34, BIM, PUMA [8].

MSA synergistically enhances the growth-inhibitory efficacy of paclitaxel in MDA-MB-231 cells. MSA enhances paclitaxel-induced apoptosis. MSA could enhance paclitaxel-mediated G2/M arrest suggests the potential of using MSA to overcome paclitaxel resistance [83].

**Figure 3:**
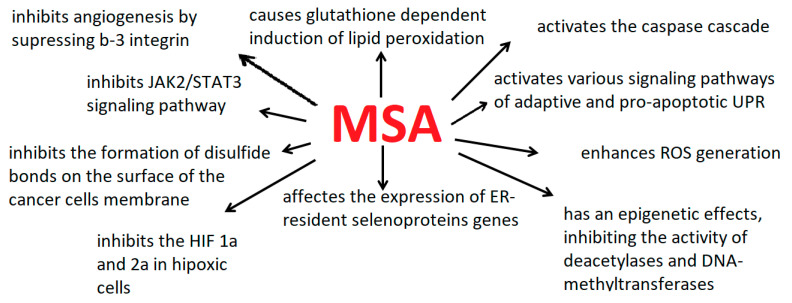
The main effects of MSA on cancer cells.

Figure 3 shows schematically the main effects of MSA on cancer cells. However, in order to understand the whole picture of the molecular mechanisms of ER-stress and apoptosis regulation in cancer cells with the active participation of MSA, it is necessary to pay special attention to the study of the effect of MSA on the expression and activity of ER-resident selenoproteins. Table 1 shows the main molecular mechanisms of the cytotoxic action of MSA on the example of various cancer cells.

## 4. Differential Expression of ER-Resident Selenoprotein Genes under ER-Stress Conditions Caused by the MSA

To date, it is known that in mammals seven selenoproteins are localized in the ER: selenoprotein M (SELENOM), selenoprotein F (SELENOF), selenoprotein T (SELENOT), selenoprotein K (SELENOK) S (SELENOS), iodothyronine deiodinase 2 (DIO2), and selenoprotein N (SELENON). The ER-resident selenoproteins, according to their structural features, are classified into families. Thus, SELENOM, SELENOF, and SELENOT belong to the family of proteins with thioredoxin-like folding, while SELENOS and SELENOK belong to the family of type III transmembrane proteins [84,85,86,87,88]. SELENON is a transmembrane glycoprotein containing an EF motif in its structure [89]. DIO2 is a dimeric type 1 membrane protein that is inactivated by ubiquitinylation [90,91].

The localization of these selenoproteins in the ER suggests their participation in the regulation of processes associated with ER stress, which has been repeatedly demonstrated by a number of works [3,4,8,10,25,92], and the expression of genes encoding these selenoproteins, and the activity of the proteins themselves, are largely regulated by various inducers of ER-stress, especially of selenium nature. However, the nature of the regulation of ER-resident selenoproteins gene expression is very ambiguous and depends not only on the nature of the source of ER stress but also on its concentration and time of treatment. This is quite logically explained by the fact that the concentration and time of treatment with one or another inducer determine the severity of ER stress, which may differ significantly not only in normal and cancer cells, but also when comparing cancer cell lines with each other. This fact has been repeatedly confirmed by tests for the viability and proliferative properties of various cell lines when cells are treated with different types of ER-stress inducers [93,94,95].

Previously, we analyzed the expression of genes of seven ER-resident selenoproteins depending on the concentration and time of treatment with MSA on cancer cells of three cell lines: DU 145-prostate carcinoma, HT-1080-fibrosarcoma, MCF7-breast adenocarcinoma [3]. We carried out a comparative analysis of the mRNA expression of selenoprotein genes by families into which they are assigned by their structural features. Thus, when comparing the expression of three selenoproteins, SELENOM, SELENOF, and SELENOT, we established the synchronous nature of the mRNA expression patterns of the SELENOT and SELENOF genes and the asynchronous expression pattern for SELENOM. Moreover, in DU 145 and MCF7 cells, a similar pattern of changes in gene expression of all three selenoproteins was observed, while for the HT-1080 line, an inverse correlation of gene expression depending on the MSA concentration compared with two other lines was established, but a synchronous pattern of SELENOT and SELENOF expression and an asynchronous one for SELENOM was always observed.

When DU 145 and MCF7 cells were treated with 0.01 μM MSA for 24 h, there was a slight decrease in the expression of SELENOM mRNA and, at the same time, an increase in the expression of SELENOT and SELENOF mRNAs; a slight increase in SELENOM mRNA expression and a decrease in SELENOT and SELENOF mRNAs expression were observed when these cell lines were treated with a higher concentration of MSA (0.1 μM) for 24 h. When MCF7 cells were exposed to 1 μM MSCs (the highest studied concentration) for 24 h, an even greater increase in the expression of SELENOM mRNA and insignificant changes in the expression of the SELENOT and SELENOF genes were observed as compared to the treatment of cells with 0.1 μM MSCs. However, when DU 145 cells were treated with 1 μM MSA for 24 h, a significant increase in the expression of the SELENOF and SELENOM genes was observed. The SELENOM gene is characterized by a direct correlation between the enhancement of mRNA expression depending on the concentration of MSA in both cell lines, while an inverse correlation was established for the SELENOF gene. A similar decrease in expression with an increase in MSA concentration was observed for the SELENOT gene in Caco-2 cells, while in A-172 cells, a twofold increase in the expression of this gene was found when cells were exposed to 0.1 μM MSA and a subsequent decrease in expression when cells were treated with 1 μM MSA.

Thus, using the example of five cancer cell lines DU 145, MCF7, A-172, Caco-2, and HT-1080, we studied, firstly, the level of SELENOM mRNA expression increases in proportion to the increase in MSA concentration in all lines. Second, the expression patterns of SELENOT and SELENOF at low MSA values (0.01 and 0.1 μM) are almost always synchronous. Third, under conditions of prolonged ER-stress induced by 1 μM MSA for 24 h, the expression levels of SELENOM and SELENOF tend to close values, as a rule, the expression is enhanced, which was shown by us on the example of four human cancer cell lines: DU 145, MCF7, A-172 and HT-1080 [3].

Taking into account the fact that SELENOM and SELENOF have a similar structure and belong to the same family, they are most likely the most active and interact at the end stages of cell apoptosis, in particular, in membrane blebbing, as it was proved for SELENOF [96]. Indirectly, the involvement of SELENOM in this process was shown by us earlier [92], since the physiological partners of SELENOM in cancer cells MCF7 and HT-1080 were cytoskeletal actin 1 and 2, which play a key role in cellular processes such as adhesion, migration, polarization, and mitosis. It is known that pathological changes in cell motility are observed during tumor transformation caused by dysregulation of the actin system, which leads to tumor invasion and metastasis.

Most likely, the high expression of SELENOT at the early stages of ER stress may indicate the participation of this selenoprotein in the degradation of misfolded proteins under ER-stress conditions. In support of this hypothesis, there is a study that shows that SELENOT is a subunit of the A-type oligosaccharyltransferase complex (OST). Despite the fact that the sequences of the two selenoproteins SELENOK and SELENOS do not have homology, the proteins have a similar domain organization and belong to the third type of transmembrane proteins [97,98]. We have previously shown that mRNA expression patterns of genes of these proteins and the quantitative content of selenoproteins in DU 145, MCF7, A-172, and HT-1080 cells change synchronously, depending on the MSA concentration. A high level of mRNA expression of both proteins, as a rule, was observed when cells were treated with 0.01 μM and 1 μM MSA, while when cells were treated with 0.1 μM MSA, their expression changed insignificantly from the control [3]. The high degree of mRNA expression and the activity of SELK and SELS proteins under conditions of adaptive UPR can be explained by the fact that both proteins interact with the components of the oligosaccharyltransferase complex (OST): Derlin-1, Derlin-2, riboforins I and II, OST48, STT3A [99,100]. In addition, both selenoproteins control the transfer of the vasolin-containing protein p97 (VCP) to the ER membrane [101,102,103,104,105,106]. These protein-protein interactions SELENOK and SELENOS indicate their involvement in the regulation of the ERAD-system, such as SELENOT, causing the degradation of proteins with incorrect folding. The high expression and activity of these selenoproteins during prolonged stress is most likely due to their antioxidant function, which is aimed at maintaining redox homeostasis in cells.

SELENON is a type II transmembrane protein whose activity largely depends on fluctuations of calcium ions with the EF-hand domain (helix-loop-helix structural domain) of selenoprotein, which consists of two alpha helices linked by a short loop region (usually about 12 amino acids) that usually binds calcium ions. [89]. It is believed that SELENON is a kind of sensor for the concentration of calcium ions in the ER lumen, which changes its oligomeric state upon ion depletion [85]. In addition, it has been shown that SELENON can play an important role in protecting cells from oxidative stress and maintaining Ca^2+^ homeostasis, interacts with the ryanodine receptor RYR1, and can neutralize hydrogen peroxide-induced inhibition (SERCA2b) [107,108]. Another ER-resident selenoprotein is DIO2, which is a dimeric type 1 membrane protein that is inactivated by ubiquitinylation [91,109,110]. DIO2 activity is regulated at different levels. This selenoprotein undergoes the reaction of ubiquitination of E3-ubiquitin ligase WSB-1 and TEB4, after which it can interact with VCP and is transferred to the cytosol for proteasome degradation [111,112,113]. When studying the changes in the mRNA expression of these two selenoproteins from the concentration of MSA, it was difficult for us to trace any regularity, as it was established for the other five ER-resident selenoproteins. In different cancer cell lines, their expression changed in different ways; for example, the expression of SELENON mRNA increased in proportion to the concentration of MSA in DU 145 and A-172 cells and decreased in Caco-2 and HT-1080 cells. In addition, in cells, MCF7 practically did not differ from control. Whereas the expression of DIO2 mRNA decreased in A-172 and MCF7 cells in proportion to the increase in MSA concentration and increased in the other three cell lines [114].

## 5. Discussion

Currently, there are many known ER-stress inducers of various nature; among them, special attention is paid to selenium-containing agents since we and other authors have repeatedly shown that even insignificant concentrations of some of them can cause not only adaptive but also prolonged ER stress leading to cell death [2,3,4,5,6,7,8,9,10]. The special interest in selenium compounds, which affect the redox processes of tumor cells, is explained by the fact that drugs based on them induce complex cascades of redox reactions, which ultimately lead to apoptosis and are multisite drugs. The development of resistance to such drugs is difficult because they have a targeted effect on highly specialized molecular targets. Thus, the antioxidant defense mechanisms of cancer cells are overloaded both by their own generation of ROS and their generation from redox-active selenium compounds, which leads to the loss of structural and functional integrity and the subsequent death of cancer cells. Such a treatment strategy can be very effective, especially when combined with monosite drugs, ROS generators, used in oncology.

This review is devoted to the study of the cytotoxic effect of a selenium-containing organic compound, methylselenic acid (MSA), on various cancer cells, on the regulation of the expression of seven ER-resident genes, and on the activation of various signaling pathways of both adaptive and prolonged ER stress caused by various concentrations of MSA. The analysis of a large number of works indicates that there is no strictly specific molecular mechanism of the cytotoxic action of this selenium-containing compound in relation to various cancer cell lines and cancer models.

Among the well-known mechanisms and regulatory pathways of the antitumor activity of MCA, the following can be distinguished: glutathione-dependent induction of lipid peroxidation [34], the inhibition PI3K/AKT/mTOR pathway and activation of FOXO proteins [54], the inhibition of the activity of deacetylases and DNA-methyltransferases [114], the activation of the Keap1/Nrf2 pathway via upregulation of miR-200a [32], the activation signaling pathways of adaptive and pro-apoptotic UPR, the down- and up-regulation of the ER-resident selenoprotein genes expression [3], the inhibitions of angiogenesis by suppressing β3-integrin and interrupting its clustering [41] and other cytotoxic effects.

In addition, using the example of various cancer cell lines, a pattern in the regulation of the expression of selenoprotein genes localized in the ER was revealed, especially three of them: SELENOM, SELENOF, and SELENOT [3]. Firstly, the level of SELENOM mRNA expression increases in proportion to the increase in MSA concentration in all lines. Second, the expression patterns of SELENOT and SELENOF at low MSA values (0.01 and 0.1 μM) are almost always synchronous. Third, under conditions of prolonged ER-stress induced by 1 μM MSA for 24 h, the expression levels of SELENOM and SELENOF tend to close values, as a rule, the expression is enhanced, which was shown by us on the example of four human cancer cell lines: DU 145, MCF7, A-172, and HT-1080. mRNA expression patterns of SELENOK and SELENOS genes of these proteins and the quantitative content of selenoproteins in DU 145, MCF7, A-172, and HT-1080 cells change synchronously, depending on the MSA concentration [3].

## 6. Conclusions 

This review provides a comprehensive understanding of the complex and controversial mechanisms of the cytotoxic action of the well-known antitumor agent of selenium nature, which has a number of significant advantages over other MSA. All these qualities make methylseleninic acid an attractive substrate for the production of anticancer drugs on its basis with a well-pronounced selective effect.

## Figures and Tables

**Table 1:** The main molecular mechanisms of regulations of MSA cytotoxicity on the example of various cancer cell lines.

Cancer Cell Line Name	Molecular Mechanisms of Regulations of MSA Cytotoxicity	Reference
A-172 (human glioblastoma)	Treatment with 1 μM MSA during 24 h reduces proliferation by 70–80% and increases the expression of mRNA of the transcription factors ATF-4 and ATF-6. Silencing of SELENOT under ER-stress induced by 0.1 μM MSA resulted in an increase in the expression of SELENOM and decreases the expression of AMFR (autocrine motility factor receptor) and RNF5 (ring finger protein 5), which are E3-ubiquitin ligases, important enzymes of the ERAD-system.	[3]
A549 (human lung cancer cells)	MSA reduced cell growth by 50% when cells were treated with 2.2 ± 0.3, 1.6 ± 0.2, and 1.3 ± 0.1 μM for 24, 48, and 72 h, respectively.	[32,54]
	Micromolar concentrations of MSA markedly inhibited the growth of A549 cells. MSA induces G1 arrest by down-regulating cyclin E1 and up-regulating p27Kip1. MSA was simultaneously shown to enhance apoptosis induction in the presence of intracellular GSH. Over 67% of cells were consecutively inhibited at the G0/G1 phase.	
	5 μM MSA attenuates the activity of glycolysis, TCA cycle, PPP, and/or nucleotide biosynthesis. MSA effects are associated with the inhibition of the Akt pathway, leading to dephosphorylation of FOXO proteins and their nuclear translocation, which in turn activate the expression of FOXO target genes. FOXO dephosphorylation and relocalization to the nucleus are early events that activate the antiproliferative response of A549 cells to MSA	
Cal27 (tongue origin, CRL-2095), SCC25 (tongue origin, CRL-1628) (head and neck squamous carcinoma cells)	Treatment with 10 μM MSA during 24 h appears to be more toxic to SCC25 compared to Cal27 cells. MSA inhibits cell proliferation by more than 90%.	[34]
	Lipid peroxidation (LPO) is an essential step in the MSA-induced toxicity of HNSCC cells. MSA sensitizes HNSCC cells to radiation and exhibits toxicity through a GSH-dependent induction of LPO. Cal27 cells treated with 10 µM MSA for 72 h were found to have 1.16 fmol lipid hydroperoxide per cell, nearly 40 times as much as untreated cells	
DU145 (human prostate carcinoma epithelial cells)	At 1 μM MSA, the viability of these cell lines decreases by 70–80%. MSA promoted the activation of the PERK signaling pathway, increases expression of apoptosis genes, as well as effector caspase-3 and inflammatory caspase-4. When DU 145 cells were treated with 1 μM MSA for 24 h, a significant increase in the expression of the SELENOF and SELENOM genes was observed.	[3]
Eca109 (human esophageal carcinoma cell line)	Treatment of cells with 20 and 40 μM MSA for 72 h reduced cell viability by only approximately 50%.	[65]
	After treatment of Eca109 cells with 20 μM MSA for 48 h cell cycle was arrested in G0/G1 phase, and the cell population was increased in the S phase. MSA significantly reduces the expression of FAL1 (focally amplified lncRNA on chromosome 1), and thus, the level of PTEN (phosphatase and tensin homolog deleted on chromosome 10) is increased.	
HUVEC (human umbilical vein endothelial cells)	A total of 10 μM MSA inhibits cell proliferation by 20% during 24 h.	[41]
	Effectively increases the adherence to collagen I and inhibits cell migration of HUVECs; down-regulates Integrin β3 and inhibits phosphorylation of AKT; disrupts the clustering of integrin β3 surface localization; inhibits VEGF-induced angiogenesis and the phosphorylation of IκBα and NF-κB, and the nuclear translocation of NF-κB	
KYSE150, KYSE180, KYSE410, and KYSE510 (ESCC-human esophageal squamous cell carcinoma cells):	MSA treatment significantly down-regulated Keap1 (Kelch-like ECH-associated protein 1), induced nuclear accumulation of Nrf2 (nuclear factor E2-related factor 2), and enhance the ARE (antioxidant response element) promoter activity and significantly induce miR-200a expression.	[31]
MDA-MB-231(human breast adenocarcinoma cells)	The viability of cells treated with 4 μM MSA for 72 h decreased by more than 40%, while simultaneously treating cells with 4 μM MSA and 10 nM paclitaxel for 72 h-by more than 80%.	[83]
	MSA synergistically enhances the growth-inhibitory efficacy of paclitaxel in MDA-MB-231 cells. MSA enhances paclitaxel-induced apoptosis. MSA could enhance paclitaxel-mediated G2/M arrest suggests the potential of using MSA to overcome paclitaxel resistance	
PANC-1, PANC-28, Colo357, Bxpc-3, HPAC (human pancreatic cancer cell) lines	PANC-1 treatment with 2.6 μM MSA for 5 d, PANC-28 treatment with 1.2 μM MSA for 3 d, Colo357 treatment with 0.6 μM MSA for 48 h, Bxpc-3 treatment with 1.15 μM MSA for 48 h, HPAC treatment with 3.7 μM MSA for 48 h resulted in a 50% decrease in cells growth.	[64,82]
	MSA induced G1 arrest and caspase-mediated apoptosis in most pancreatic cancer cell lines and manifested a rapid G2 arrest in the PANC-1 and PANC-28 cell lines. MSA induced G1 arrest in Colo357, Bxpc-3, HPAC cells at 12, 24, and 48 h. A total of 7.5 μM MSA induced a modest 2.2-fold of apoptotic fragmentation in PANC-1 cells compared to control.	
	When PANC-1 cells were treated with 1 μM MSA for 72 h, a decrease in cell viability was observed only by 20%, while when treated with 50 μM MSA, by more than 90%.	
	MSA induced entosis by cell detachment through downregulation of cell division control protein 42 homologs (CDC42) and its downstream effector β1-integrin (CD29). Treatment with MSA led to a unique phenotype, characterized by changes in morphology and cell detachment from the culture plate prior to cell death.	
PEL (primary effusion lymphoma)	Treatment with 30 μM MSA during 24 h reduces proliferation by 70–80%.	[8]
	MSA induces pro-apoptotic UPR through transcriptional activation of pro-apoptotic genes, CHOP, Bim, and Puma, via the activation of caspases, induces oxidative stress but not lytic replication	
4T1 (mouse malignant breast cancer cells)	MSA significantly induces apoptosis of these cancer cells by activating Bax, caspase-3, PARP. In addition, MSA is able to inhibit tumor angiogenesis by reducing the expression of vascular endothelial growth factors VEGF and Ang-2 in mammary cells of dogs and mouse models. In addition, this series of experiments showed that MSA inhibited the JAK2/STAT3 signaling pathway.	[59,60]
WM1552c, UKRV, Colo875 (human melanoma cell lines), SK-BR-3, BT-474 (human mammary carcinoma cell lines), B16F10 (mouse skin melanoma cells)	Treatment with MSA increased the MHC class I (major histocompatibility complex surface) expression levels in all the tested tumor cell lines. MSA partially mimics IFNγ signaling, such as the upregulation of STAT1(Stat1 signal transducer and activator of transcription 1), JAK1 (janus kinase 1), IRF1, IRF5, IRF7, and IRF9 (interferon-regulated factor1, 5, 7 and 9) on the mRNA and/or protein expression levels. In addition, MSA treatment leads to activation of the transcription factor Nrf2.	[62]
HNSCC (human head and neck squamous cell carcinoma),	MSA effectively inhibits the HIF-1α in hypoxic cells, while PHD 2 and PHD 3, on the contrary, were activated, which was demonstrated in HNSCC cells.	[73]
RC2 and 786-0 (clear cell renal cell carcinoma)	The resistance of these cancer cells, overexpressing the HIF-1α under hypoxia, to SN38-the active metabolite of irinotecan, was reduced after MSA treatment. Similar synergistic activity of MSA in combination with docetaxel was shown in a model of prostate cancer cells, which can also be explained by the inhibition of HIF-1α by the activation of PHDs.	[74,75,76,77]
(PC-3 and PC-3M, PAIII and DU145 (human prostate cancer cells)	Treatment of clear cell renal cell carcinoma (RC2 and 786-0) with a pharmacological dose of MSA (10 μM) promoted inhibition of constitutively expressed transcription factors HIF-1α and HIF-2α in RC2 and 786-0 cells, respectively.	[78]
	MSA inhibits the expression and activity of HIF-1α in invasive rat and human prostate cancer cells [79]. Thus, the treatment of highly aggressive human prostate cancer cell lines (PC-3 and PC-3M) with MSA led to significant inhibition of growth and induction of apoptosis, and MSA has a stronger effect on cells under hypoxia than under normoxia, especially at a physiological dose of MSA (5 μM).	[79]

## Data Availability

The data presented in this study are available on request from the corresponding author.
